# The Increased Early Onset Colorectal Cancer in South East Scotland Is Indicative of a Wider UK Problem

**DOI:** 10.3390/cancers17121913

**Published:** 2025-06-09

**Authors:** Adam D. Gerrard, Hannah Garside, Yasuko Maeda, Evropi Theodoratou, Malcolm G. Dunlop, Farhat V. N. Din

**Affiliations:** 1 Cancer Research UK Scotland Centre, Institute of Genetics and Cancer, The University of Edinburgh, Edinburgh EH4 2XU, UK; adam.gerrard@ed.ac.uk (A.D.G.);; 2 Department of Colorectal Surgery, Western General Hospital, Edinburgh EH4 2XU, UK; 3 School of Medicine, Dentistry and Nursing University of Glasgow, Glasgow Q12 8QQ, UK; 4 Department of Surgery, Queen Elizabeth University Hospital, Glasgow G51 4TF, UK; 5 Centre for Global Health, Usher Institute, The University of Edinburgh, Edinburgh EH8 9YL, UK; 6 UK Colon Cancer Genetics Group, Medical Research Council Human Genetics Unit, Medical Research Council Institute of Genetics & Cancer, Western General Hospital, The University of Edinburgh, Edinburgh EH4 2XU, UK

**Keywords:** early onset colorectal cancer, EOCRC

## Abstract

**Background and Aims:** Colorectal cancer (CRC) in those younger than age 50 (early onset colorectal cancer: EOCRC) is reported to be increasing. We aimed to determine whether trends in incidence regionally were being reproduced across the UK. We investigate clinically important differences between CRC cases older (LOCRC) and younger than 50 years of age. **Methods:** The South East Scotland Cancer Network (SCAN) database was interrogated for CRC within NHS Lothian (2002–2019). Public Health Scotland and the Office for National Statistics (ONS) publish cancer data for Scotland, England, and Wales (1993–2019). Population figures were gathered from National Records of Scotland and ONS data. Joinpoint analysis was performed to assess significant Annual Percentage Change (APC) in CRC incidence trends. **Results:** In NHS Lothian, there has been a steady significant increase in EOCRC with an APC of 2.87 (*p* < 0.05) cumulating with an incidence rate of 10.5/100,000 in 2019. This was most evident in males and those aged 30–39. Across Scotland, England, and Wales, there have been similarly significant increasing trends of EOCRC. EOCRC was more likely to be referred as routine priority (23.2% vs. 4.0%, *p* < 0.001) and diagnosed at a later stage (AJCC stage 3/4), with 62.8% compared with 52.5% in the LOCRC, *p* < 0.001. There was better overall 5-year survival for EOCRC (*p* = 0.005). **Conclusions:** The incidence of EOCRC is increasing within NHS Lothian and across the UK. Awareness of EOCRC is essential to improve the current problems with delayed diagnosis and advanced stage disease.

## Simple Summary

Early-onset colorectal cancer (EOCRC), which occurs in people under 50, is rising in South East Scotland and across the UK. This study examined cancer registry data from NHS Lothian and national sources between 1993 and 2019. It found a steady increase in EOCRC rates, particularly among men and those aged 30–39. Compared to older patients (LOCRC), younger patients were more likely to be diagnosed at a later stage and after routine (non-urgent) referrals. The findings suggest that EOCRC is not just a local issue but a UK-wide trend. The study highlights the need to raise awareness, adapt referral pathways, and consider tailored screening or diagnostic tools (like FIT testing) for younger adults. Addressing delays in diagnosis could help improve outcomes for this growing group.

## 1. Introduction

Worldwide, colorectal cancer (CRC) is the second most fatal cancer [1]. Whilst there is considerable variation in incidence worldwide, high-incidence locations have long been associated with environmental and lifestyle factors. Recent trends point to stabilisation of overall incidence and indeed a decrease in high-income countries [2,3]. Parallel reports from varied geographical locations suggest that there is an increase in early onset colorectal cancer (EOCRC) [4]. EOCRC is defined as CRC occurring in patients under the age of 50 years old and since 1995, the number of people diagnosed with EOCRC has been reported to be increasing by 2% per year, accounting for up to 10% of all CRC [5,6]. EOCRC is hypothesised to be biologically different to later onset colorectal cancer (LOCRC) [7] and this coupled with often delayed presentation [8] cumulates in more advanced disease at diagnosis [9] with unfavourable pathology [10]. Hence, the underlying basis for the reported increase in EOCRC has important implications for clinical practice.

Hereditary predisposition syndromes only account for ~25–30% of cases, highlighting that the majority are sporadic [11]. Several risk factors have been hypothesised including diets high in red or processed meats, dietary additives, glutamate and increased antibiotic use [12], obesity, physical inactivity, microbiome imbalance [13], and male sex [6,14]. However, many of these factors are also associated with CRC formation later in life as exposure to risk accumulates. This raises the possibility of a birth cohort effect with certain exposures, yet unclear, being responsible for the increase in CRC at younger ages. Factors proposed to be implicated in the increase include fertility treatments, caesarean section births, maternal obesity, high birth weights, early exposure to antibiotics, environmental contaminates, and chronic stress [15].

Early diagnosis clearly impacts better survival outcomes for all CRCs [16]. Acknowledging the EOCRC evidence base, the American Cancer Society and US Preventive Services Task Force recommended screening at age 45 [17]. Bowel screening programmes in the UK do not target those < 50 years and a considerable increase in colonoscopy capacity would be required to lower the screening age. Whilst some studies have suggested that EOCRC is more common in the left colon and rectum [18], this does not translate to any distinguishing symptom that aids early diagnosis. Therefore, early detection hinges on timely presentation to primary care and prompt referral, which is impeded by the non-specific nature of symptoms and the low index of suspicion especially in younger age groups. The culmination is that EOCRC is detected at a later stage of the disease with poorer survival. Hence, the potential increase in EOCRC represents a clear area of unmet need to understand biological drivers and develop strategies that aid early detection.

The aim of this study was to determine whether there had been an increase In EOCRC within the regional population and to compare the population trends with Scotland as a whole and England and Wales. Diagnostic features and survival trends of CRC cases within NHS Lothian were assessed for clinically important differences amongst EOCRC that may aid the development of earlier diagnostic pathways.

## 2. Methods

### 2.1. Data Collection

Publicly available National Records of Scotland data was accessed for mid-year population estimates within NHS Lothian and Scotland. This is available by age category, resulting in population sizes age 20–49 (EOCRC) and 50 years and over (LOCRC) [19]. The South East Scotland Cancer Network (SCAN) contains CRC data from the NHS Lothian Health Board (2002 onwards), which provides care for nearly 900,000 people, with colorectal service being delivered at a single tertiary centre diagnosing around 500 cases of CRC per year. This data includes patient demographics, tumour location, stage, treatment, and survival. Within this data, a nested case–control study was formed using SCAN data, where all the cases of EOCRC were compared with a subgroup of matched LOCRC controls. The cases were matched across the whole EOCRC cohort using sex, postcode, and year of diagnosis. These matched case–controls were used to assess referral priority from primary care and tumour pathology findings. Assessment of faecal immunochemical test (FIT) in those under the age of 50 and with rectal bleeding was made from sub-analysis of study data previously published [20].

Similar population data is available for England and Wales via the Office for National Statistics. Country level cancer data (1993 onwards) for Scotland is provided through Public Health Scotland [21] and the Office for National Statistics in England and Wales [22]. Age-specific cancer details were not available for Northern Ireland.

### 2.2. Data Analysis

Trends in cancer diagnosis were estimated using Joinpoint regression analysis. The Joinpoint Regression Program [23,24] (Version 4.9.1.0, April 2022) tests rate change trends in data overtime. Each Joinpoint represents a statistically significant change in trend, with the analysis testing from zero to a maximum four Joinpoints. The annual percentage change (APC) for each trend is identified and considered significant when *p* < 0.05.

Continuous data were compared by Mann–Whitney U test and categorical data using *X*^2^ test or Fisher’s exact test as appropriate. Kaplan–Meier curves and Cox proportional hazards regression analysis were created to assess survival. The Scottish Index of Multiple Deprivation (SIMD) tool was used to assess levels of socioeconomic deprivation of patients within the study. This creates decile ranks from most (1) to least (10) deprived areas from postcodes. Data analysis was performed using R V 4.0.5 [25] with associated packages and GraphPad Prism V10.4.1 [26].

## 3. Results

### 3.1. Trends in CRC Within NHS Lothian: Increase in EOCRC

Between 2002 and 2019, 8348 CRCs were diagnosed within NHS Lothian, median age 71 years (IQR 63–80), and 3689 (44.2%) were female. Of the CRCs, 469 (5.6%) were EOCRC in patients under the age of 50 years. The median age at diagnosis of EOCRC was 45 years (IQR 38–48), compared with 73 years (IQR 64–80) in the LOCRC. Two hundred and thirty of the EOCRCs were female, which was a significantly greater proportion than in the LOCRC (49.0% vs. 43.9%, *p* = 0.028).

Over the study period, the average population of NHS Lothian, aged 20 years and over, was 652,291 persons, with 375,031 (57.5%) of these under 50 years old, and 52.2% were female. The population increased steadily during the study from 600,185 in 2002 to 719,245 in 2019, and aged, with 59.0% under 50 in 2002 versus 55.7% in 2019 (Supplementary Figure S1).

**Figure 1:**
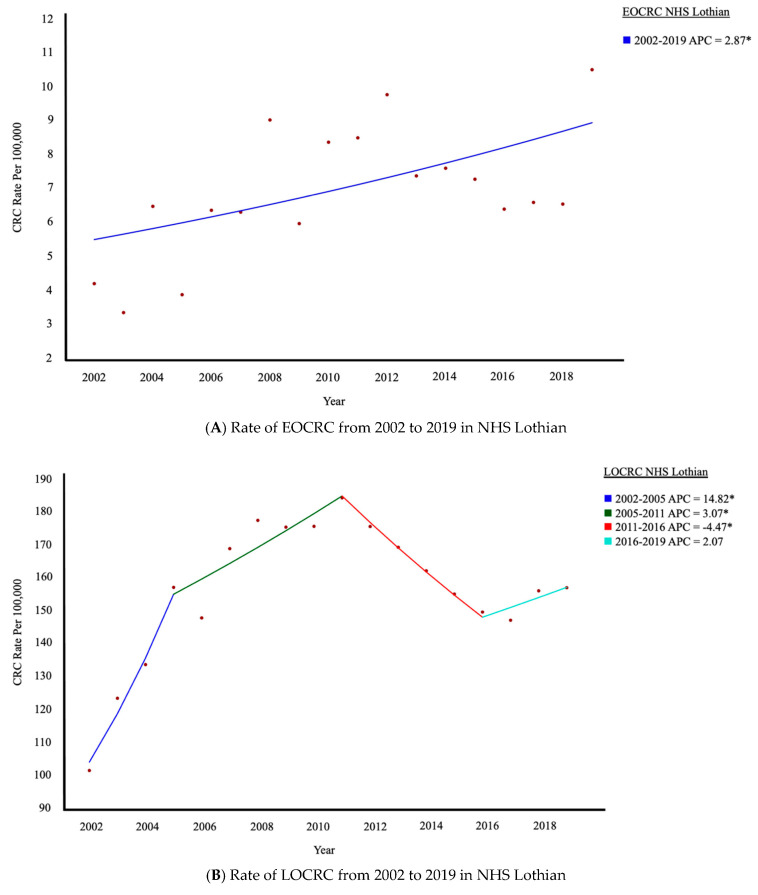
Rates of EOCRC and LOCRC within NHS Lothian and the trends of the Annual Percentage Change. * Indicates that the Annual Percentage Change (APC) is significantly different from zero at the alpha = 0.05 level.

The rate of EOCRC within the NHS Lothian population has shown a steady significant increase since 2002 from 4.2/100,000 to 10.5/100,000 (Figure 1). LOCRC rates increased up to 2011 before significantly decreasing until 2016. These trends observed in LOCRC are likely influenced by the implementation of the Scottish Bowel Screening Programme (SboSP) which invites those aged 50 and over to participate. In the early years, this would see more CRCs diagnosed as they are identified through the SboSP, but in later years, the incidence falls as a result of preventive polypectomies.

**Figure 2:**
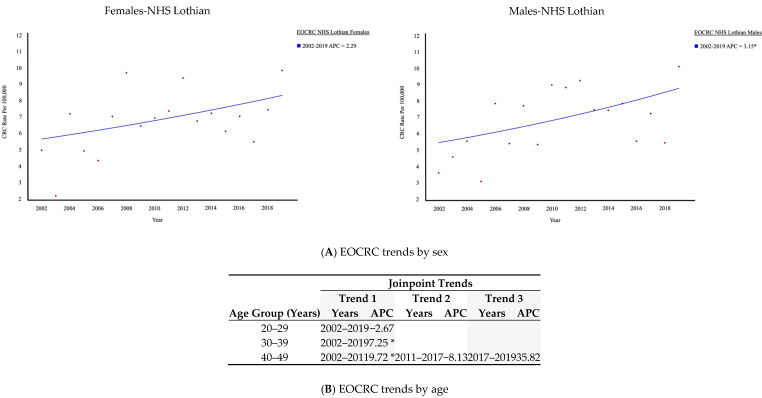
EOCRC trends within NHS Lothian by (**A**) sex and (**B**) age distribution. * indicates that the Annual Percentage Change (APC) is significantly different from zero at the alpha = 0.05 level.

Whilst there is an upward trend in EOCRC in females, it is the significantly increasing rate in males that is driving the observed rise in EOCRC (Figure 2A). When broken down into age deciles, there has been no significant change in EOCRC rates for those in their 20s; ages 30–39 years have seen a constant significant rise in EOCRC, whilst in those aged 40–49, there was a steeply increasing rate between 2002 and 2011 with a significant APC of 9.72, and non-significant fluctuation since (Figure 2B).

A potential explanation for a perceived rise in EOCRC would be due to lead time bias generated through earlier diagnosis of CRC around the arbitrary age of 50. This does not appear to be the cause of the increasing trend in EOCRC diagnosis (Table 1). NHS Lothian, Scotland, and Wales have not observed an increase in CRC in those aged 45–49, with NHS Lothian and Scotland seeing an increase in those aged 50–54. This dispels the notion that the same CRCs are being diagnosed earlier and creating the illusion that EOCRC is on the rise. In England, there is a rise in the 45–49 year olds but no reciprocal decrease is seen in those 50–54, where the rate continues to increase albeit non-significantly.

### 3.2. Comparison of EOCRC Trends in NHS Lothian to Other Areas of UK

**Figure 3:**
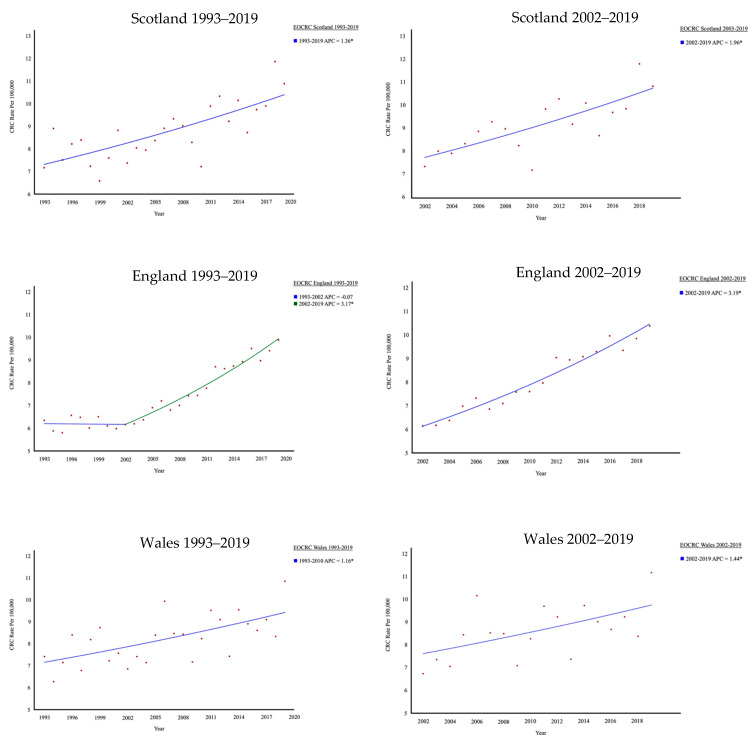
Rates of EOCRC in Scotland, England, and Wales between 1993 and 2019 and 2002 and 2019. * indicates that the Annual Percentage Change (APC) is significantly different from zero at the alpha = 0.05 level.

Across the same period of 2002–2019, Scotland has also experienced a steady significant increase in the rate of EOCRC, which also holds true when the inclusion period is extended to 1993 (Figure 3). The average rates of EOCRC in the last 5 years are higher across Scotland compared with NHS Lothian (10.2/100,000 vs. 7.5/100,000), which is a reflection of the population’s socioeconomic make up, where NHS Lothian, despite being the second largest population, accounts for less than 10% of the most deprived areas in Scotland [27]. A similar increase is observed in England and Wales, with the increase incidence of EOCRC occurring most rapidly in England. This may be due to a seemingly delayed take off in the increased trend of EOCRC in England, which on review from 1993, did not start until 2002, in contrast to Scotland and Wales, which have seen a more consistent rise.

LOCRC in Scotland sees the same significant decline following the introduction of the SBoSP as observed within NHS Lothian (Supplementary Figure S2). However, the previous increase in diagnosis across 2002–2005 in NHS Lothian is not replicated across Scotland where there was fluctuation but no significant trend in the rate of LOCRC. England and Wales show a similar pattern of LOCRC where previously increasing trends declined in the years after bowel screening was introduced.

### 3.3. Clinical Features of EOCRC Diagnosed in NHS Lothian

A subset of the CRCs diagnosed in NHS Lothian between 2006 and 2019 was reviewed (*n* = 7014). Four hundred and six (5.8%) were cases of EOCRC, which were more likely to be in the rectosigmoid and rectum, have a more advance stage at diagnosis, and be diagnosed following a routine referral from primary care (Table 2).

Comparing the EOCRC with their matched LOCRC, there was more EOCRC with symptoms of rectal bleeding (37.9% vs. 22.2%, *p* < 0.001). This presents a major challenge in primary care, as an analysis of GP referrals over 34 consecutive months to secondary care with symptoms suspicious of CRC shows that the prevalence of rectal bleeding is high generally and greater in those under 50 (64.2% vs. 35.1%, *p* < 0.001) [20].

### 3.4. FIT in Patients Under 50 Years Old

Differentiating between those under 50 years of age with rectal bleeding that might have EOCRC compared with the vast majority that will have self-limiting outlet bleeding is key to ensuring that the correct patients can be referred urgently without overwhelming the referral pathways. Faecal immunochemical testing (FIT) may offer an objective means of prioritising those under 50 years old regardless of symptoms. Between January 2019 and July 2022, symptomatic referrals to our tertiary colorectal centre have been sent FIT prior to investigation. In total, 7430 patients have been sent FIT (overall CRC prevalence 3.4%) of whom 14.2% were under the age of 50. Seven patients under the age of 50 had CRC having completed FIT, five (71.4%) had a symptom of rectal bleeding, at a threshold of 10 µg Hb/g, and no case of CRC was missed.

**Figure 4:**
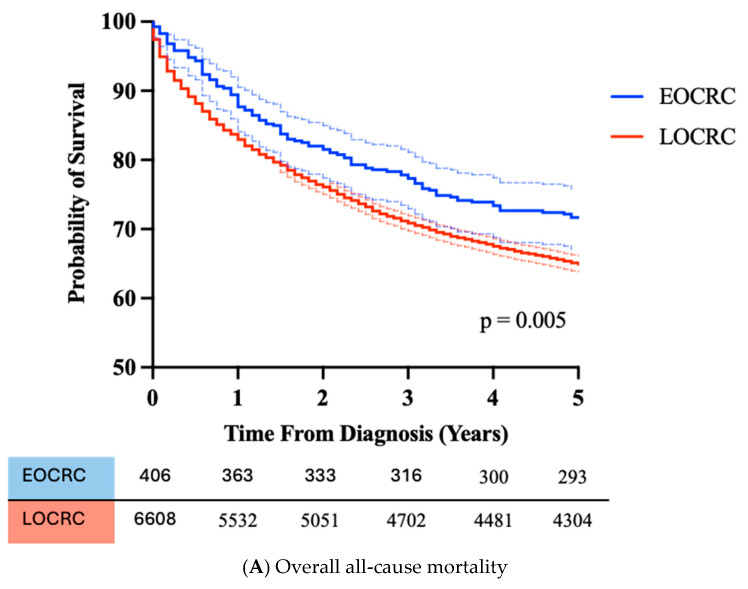

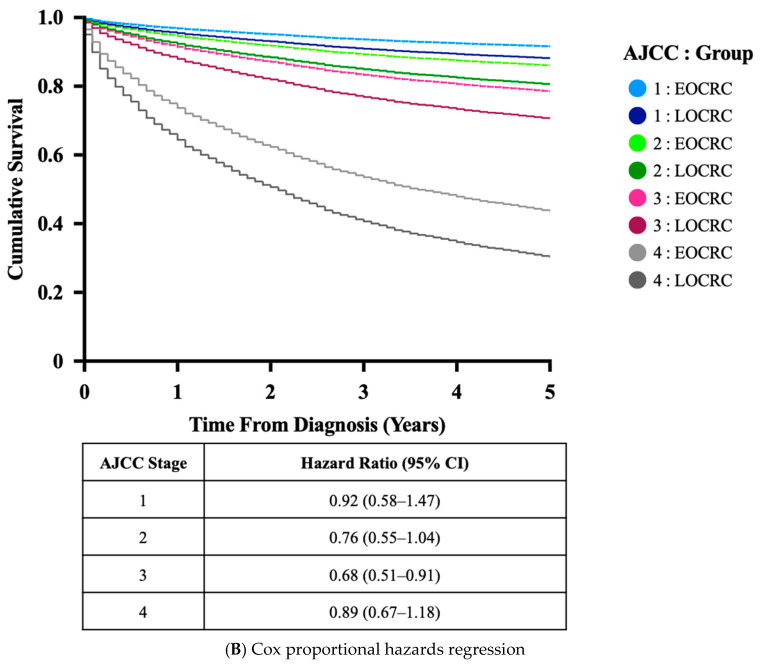
All-cause mortality for EOCRC (n = 406) and LOCRC (n = 6608) diagnosed in NHS Lothian (2006–2019) (**A**). All-cause mortality Cox proportional hazards regression analysis for AJCC stage (**B**). AJCC staging available for 406 EOCRC and 5896 cases of LOCRC.

Overall, all-cause mortality at five years for EOCRC was 28.3% compared with 35.0% for LOCRC (*p* = 0.005). Kaplan–Meier survival analysis for all-cause mortality (n = 7014) is shown in Figure 4A. AJCC stage data was available for all EOCRC and for 94.9% of LOCRC. After exclusion of cases where patients were palliated without completed staging, Cox proportional hazards regression analysis was performed to investigate the relationship between AJCC stage and EOCRC vs. LOCRC on survival (Figure 4B). The hazard ratio of mortality was significantly better in EOCRC in AJCC stage 3 CRC. Interestingly, at 2 years, for AJCC stage 4 CRC, EOCRC does have a survival advantage (*p* = 0.022), which is subsequently lost by 5 years (Supplementary Figure S3).

The occurrence of genetic conditions known to be high risk for CRC development was studied in each cohort. As expected, there was significantly more CRC in the EOCRC group with cancers related to familial adenomatous polyposis (FAP), Lynch syndrome, or MUTYH than LOCRC (18/406 vs. 27/6608, *p* < 0.001). The 18 EOCRC patients had their genetic condition diagnosed following genetic testing triggered by the CRC diagnosis (Supplementary Table S1). Eight (29.6%) of the LOCRCs with associated high-risk genetics were known to have pathological mutations prior to the development of CRC, with six of these being diagnosed within surveillance protocols.

## 4. Discussion

This study confirms that there has been a steady significant increase in the rate of EOCRC within our health board. The upward trend in EOCRC is also observed across Scotland, England, and Wales (Figure 3). This observation is in keeping with several recent studies, reporting substantial increases in the rate of EOCRC diagnosis [5,13,28]. The rise in EOCRC within NHS Lothian was being driven by males and those aged between 30 and 39 years. It has been previously reported in England that those aged 20–29 had the most sustained increase in diagnosis, with no difference between sexes [29]. The increase in EOCRC is not due to a shift towards earlier diagnosis around the age of 50, as there was neither a substantial increase in the rate of those just under 50 (45–49 years) or a reciprocal decrease in 50- to 54-year-olds. Trends for LOCRC all follow a similar pattern that appears to coincide with the inception of national bowel screening across the older population. Locally, observed patterns of LOCRC are likely influenced by the implementation of the Scottish Bowel Screening Programme (SBoSP) in 2009, which invites those aged 50 and over to participate. In the early years, this would see more CRCs diagnosed as they are identified through the SBoSP, but in later years, the incidence falls because of preventive polypectomies (Figure 1B).

Of the EOCRC diagnosed in this study within NHS Lothian, there were more left sided cancers, including rectal cancers, and these were diagnosed at a later stage and with poorer pathological features than the LOCRC. These are features of EOCRC reported in the literature [30,31], and are consistent findings, suggesting that the natural history and pathogenesis of EOCRC may differ from LOCRC [32]. Despite the later stage of diagnosis and poorer pathological features, patients with EOCRC have equal or better five year survival outcomes than LOCRC. This likely reflects the superior physiological reserve, fewer co-morbidities, and less frailty that younger people have. However, as demonstrated in stage 4 disease, this may confer an early survival benefit as observed at 2 years, but by 5 years, this is lost, ultimately due to the biology of stage 4 CRC.

Patients with EOCRC report difficulties receiving timely diagnosis [33]. In this study, EOCRC patients were more likely to have low routine priority referrals to secondary care, which inherently increases waiting time to diagnosis and may impact poorer pathology. Symptoms suggestive of colorectal cancer are vague, but faecal immunochemical testing can help to objectively select younger patients for urgent referral [34]. Studies in the USA have shown the incidence of EOCRC to be greatest in the ages of 45–49 [35], and have modelled improved screening outcomes from lowering the age of invitation to 45 [36]. Within the current resource-limited healthcare system such as in the UK, this would be unlikely to be feasible given that endoscopy services are already stretched to provide testing for screening positives, symptomatic referrals, and surveillance patients including those at high-risk.

There are limitations to this research. There were data that were unavailable for analysis and further data that could not be collected due to them not being routinely recorded, surrounding ethnicity, BMI, and co-morbidities. The lengths of the study inclusion were determined by the available data in SCAN and in the publicly published datasets. A total of 5.1% of the LOCRC staging data was not available and had to be excluded from Cox regression analysis. Data surrounding inflammatory bowel disease (IBD) was also unavailable. IBD has been shown to be a risk factor for EOCRC development [37], and IBD-associated EOCRC has high rates of metastasis, poorer histological outcomes, and increased mortality [38]. The main strengths of the study are the total capture of colorectal cancer cases over a seventeen-year period in NHS Lothian from SCAN data and twenty-six years for country-wide data in Scotland, England, and Wales.

The impact of this study is to raise awareness of EOCRC, to improve the referral pathways, and reduce the delays to diagnosis resulting in earlier stage and favourable pathological features. EOCRC is increasing across the UK and around the world. Service planning must be adapted to reflect colorectal cancer in those under 50 years old, which is a growing issue. It is possible that the COVID-19 pandemic will have a disproportionate effect on those under 50 with CRC, where those with the highest priority of referral received investigation with the limited resources.

## 5. Conclusions

Awareness of CRC as a differential diagnosis in those under 50 is key, as there are improvements to be made in appropriate priority of referrals, diagnostic times, and cancer stage at diagnosis.

## Figures and Tables

**Table 1:** Joinpoint analysis of CRC diagnosis around threshold for EOCRC vs. LOCRC.

		Joinpoint Trends					
		Trend 1		Trend 2		Trend 3	
	Ages	Years	APC	Years	APC	Years	APC
NHS Lothian	45–49	2002–2019	2.71				
	50–54	2002–2008	13.17 *	2008–2019	−1.42		
Scotland	45–49	1993–2019	0.26				
	50–54	1993–2019	0.71 *				
England	45–49	1993–2019	0.46*				
	50–54	1993–1999	0.3	1999–2002	−3.27	2002–2019	0.86 *
Wales	45–49	1993–1999	0.63				
	50–54	1993–1999	0.32				

* Indicates that the Annual Percentage Change (APC) is significantly different from zero at the alpha = 0.05 level.

**Table 2:** Comparison of EOCRC and LOCRC diagnosed within NHS Lothian.

	EOCRC	LOCRC	*p*-Value
Number of CRCs (%)	406 (5.8)	6608 (94.2)	
Median Age (IQR)	45 (39–48)	73 (64–80)	
Sex, F (%)	195 (48.0)	2906 (44.0)	0.111
Mean SIMD (SD)	6.4 (3.0)	6.3 (2.9)	0.509
*Method of Diagnosis (%)*			
Primary Care	212 (52.2)	3011 (45.6)	0.010
Emergency Presentation	115 (28.3)	1156 (17.5)	0.004
Screening Service	2 (0.4)	1044 (15.8)	<0.001
Incidental Finding	41 (10.1)	560 (8.5)	0.272
Other	36 (8.9)	837 (12.7)	0.024
*Primary Care Referral Priority (%)*			
Routine	49 (23.2)	7 * (4.0)	<0.001
Urgent	92 (43.5)	67 * (38.7)	0.403
USOC	71 (33.3)	99 * (57.2)	<0.001
*CRC Location (%)*			
Right Side	123 (30.3)	2480 (37.5)	0.004
Left or Rectal	272 (67.0)	4001 (60.6)	0.010
Left Side to Rectosigmoid	135 (33.3)	2126 (32.2)	0.662
Rectosigmoid or Rectum	137 (33.7)	1875 (28.4)	0.020
Information Not Available	11 (2.7)	127 (1.9)	
*CRC Stage (%)*			
Early (AJCC 1 and 2)	151 (37.2)	2800 (42.4)	
Late (AJCC (3 and 4)	255 (62.8)	3096 (46.9)	<0.001
Palliation Without Staging	0	374 (5.7)	
Information Not Available	0	338 (5.1)	
*CRC Pathology (%)*			
Differentiation			
Poor	89 (21.9)	53 * (13.1)	0.001
Moderate	214 (52.7)	213 * (52.5)	0.999
Well	9 (2.2)	8 * (2.0)	0.999
Information Not Available	94 (23.2)	132 * (32.5)	
Signet Cell Formation	25 (6.2)	8 (2.0)	0.004
5 Year all-cause mortality	28.3%	35.0%	0.005

SIMD: Scottish Index of Multiple Deprivation; USOC: Urgent Suspicion of Cancer; * Matched Controls; AJCC: American Joint Committee on Cancer.

## Data Availability

Summarised anonymised data will be made available on request.

## Supplementary Materials

The following supporting information can be downloaded at: https://www.mdpi.com/article/10.3390/cancers17121913/s1. Figure S1: NHS Lothian population by age 20–49 and 50 years and over, with the proportion of the population in each group over time; Figure S2: Rates of LOCRC in Scotland, England and Wales between 1993–2019 and 2002–2019; Figure S3: 2 Year all-cause mortality in Stage 4 CRC; Table S1: High-risk colorectal cancer genetic conditions associated with cases of EOCRC and LOCRC in NHS Lothian.
