# Supplementary material for: The Increased Early Onset Colorectal Cancer in South East Scotland Is Indicative of a Wider UK Problem
Source: Cancers (Basel). 2025 Jun 9;17(12):1913. doi: 10.3390/cancers17121913 (PMC12191209; doi:10.3390/cancers17121913)
Supplement: Supplementary file 1 [file cancers-17-01913-s001.zip › cancers-3596459-supplementary.pdf]

Supplementary Materials Index

Figure S1: pg.2
NHS Lothian population by age 20-49 and 50 years and over,
with the proportion of the population in each group over time

Figure S2: pg.3
Rates of LOCRC in Scotland, England and Wales
between 1993-2019 and 2002-2019

Figure S3: pg.4
2 Year all-cause mortality in Stage 4 CRC

Table S1: pg.5
High-risk colorectal cancer genetic conditions
associated with cases of EOCRC and LOCRC in NHS Lothian

Supplementary Figure S1: NHS Lothian population by age 20-49 and 50 years and over, with the proportion of the population in each group over time

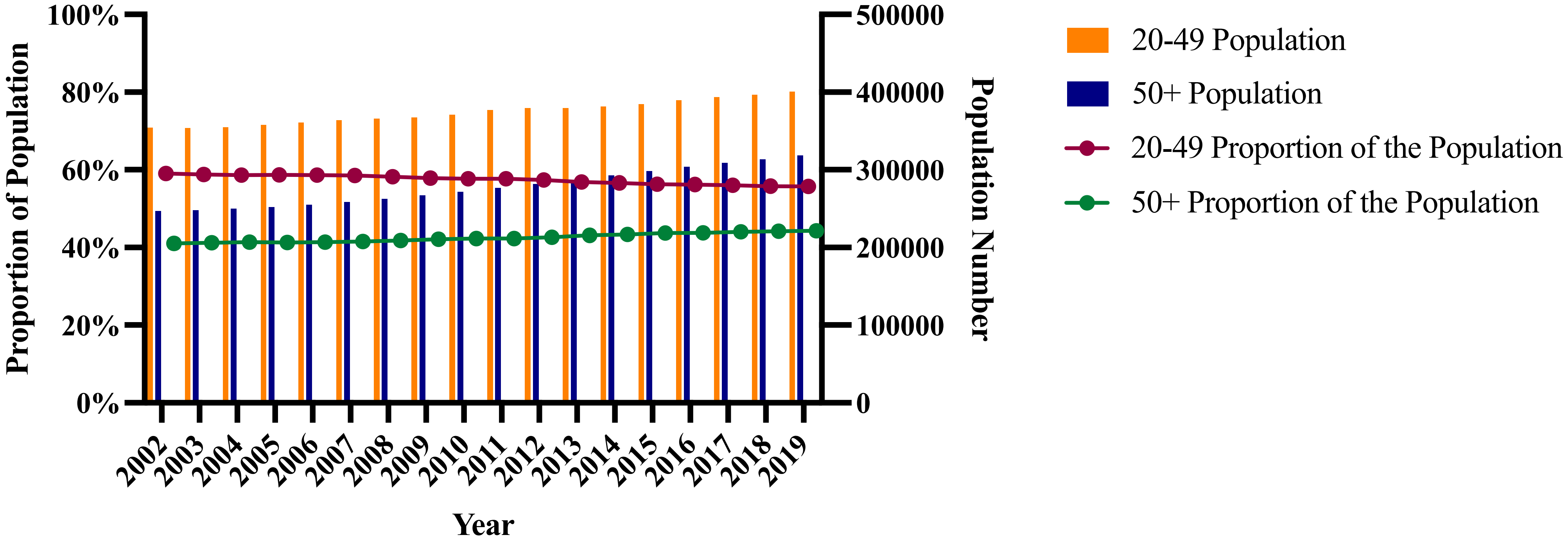

Supplementary Figure S2: Rates of LOCRC in Scotland, England and Wales between 1993-2019 and 2002-2019

*Indicates that the Annual Percentage Change (APC) is significantly different from zero at the alpha = 0.05 level

Scotland 1993-2019

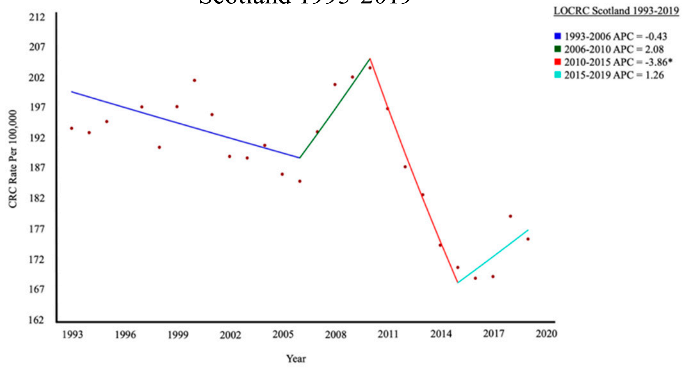

Scotland 2002-2019

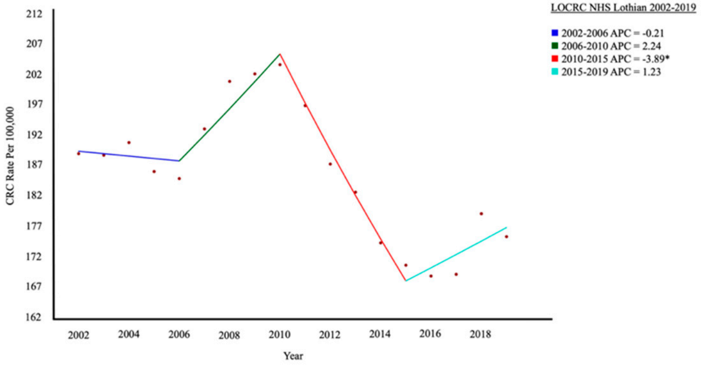

England 1993-2019

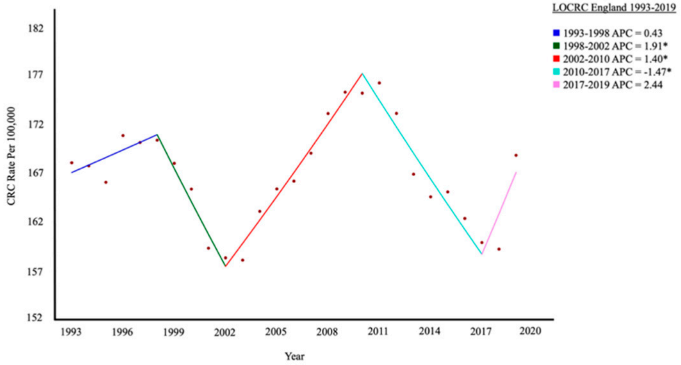

England 2002-2019

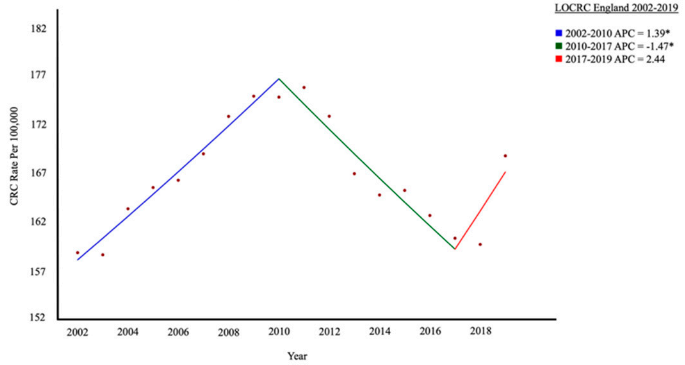

Wales 1993-2019

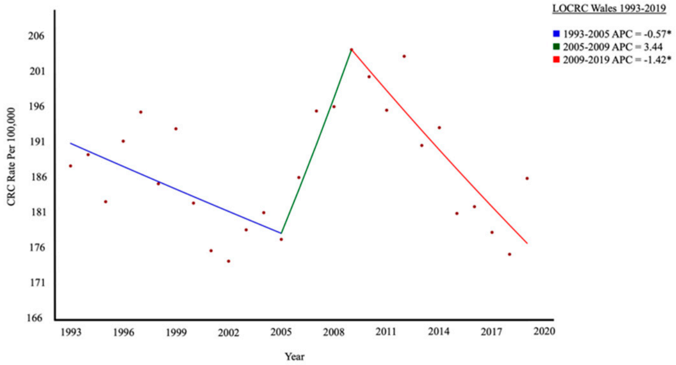

Wales 2002-2019

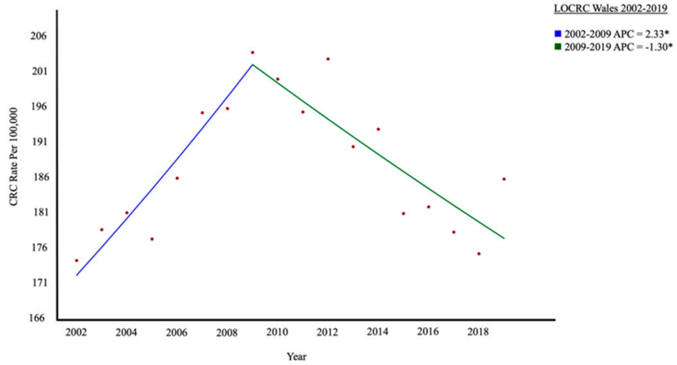

Supplementary Figure S3: 2 Year all-cause mortality in Stage 4 CRC

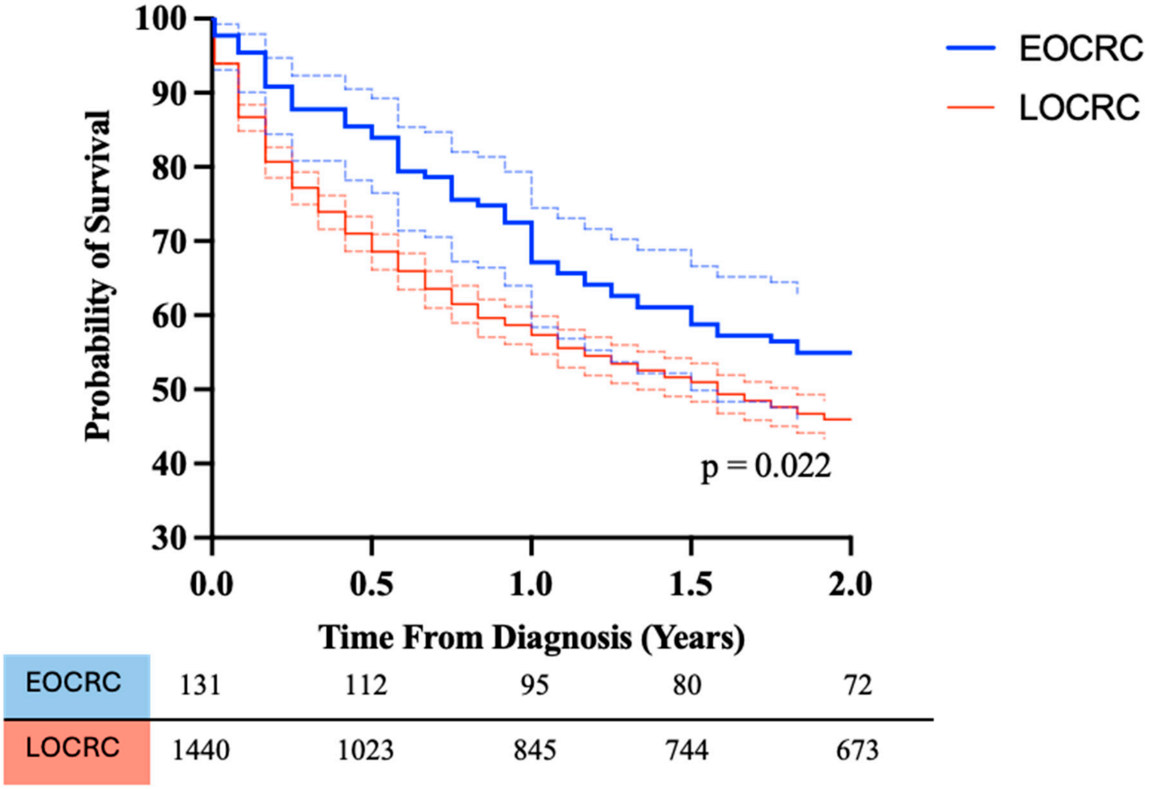

EOCRC	131	112	95	80	72
LOCRC	1440	1023	845	744	673

Supplementary Table S1: High-risk colorectal cancer genetic conditions associated with cases of EOCRC and LOCRC in NHS Lothian

Genetic Condition	EOCRC	LOCRC
Lynch Syndrome	16 (3.9%)	22 (0.3%)
FAP	2 (0.5%)	3 (0.05%)
MUTYH	0	2 (0.03%)

FAP; Familial Adenomatous Polyposis, MUTYH; MUTYH-associated polyposis, Percentages are prevalence of genetic condition to CRC diagnosed in EOCRC or LOCRC
